# Statin Treatments And Dosages In Children With Familial Hypercholesterolemia: Meta-Analysis

**DOI:** 10.5935/abc.20180180

**Published:** 2018-12

**Authors:** Graciane Radaelli, Grasiele Sausen, Claudia Ciceri Cesa, Francisco de Souza Santos, Vera Lucia Portal, Jeruza Lavanholi Neyeloff, Lucia Campos Pellanda

**Affiliations:** 1 Instituto de Cardiologia - Fundação Universitária de Cardiologia - IC/FUC, Porto Alegre, RS - Brazil; 2 Pontifícia Universidade Católica do Rio Grande do Sul (PUCRS), Porto Alegre, RS - Brazil

**Keywords:** Statins, Hydroxymethylglutaryl-CoA Reductase Inhibitors, Hypercholesterolemia Type II/genetic, Children, Meta-Analysis

## Abstract

**Background:**

Children with familial hypercholesterolemia may develop early endothelial damage leading to a high risk for the development of cardiovascular disease (CVD). Statins have been shown to be effective in lowering LDL cholesterol levels and cardiovascular events in adults. The effect of statin treatment in the pediatric population is not clearly demonstrated.

**Objective:**

To systematically review the literature to evaluate the effects of different statins and dosages in total cholesterol levels in children and adolescents with familial hypercholesterolemia. We also aimed to evaluate statin safety in this group.

**Methods:**

PubMed, EMBASE, Bireme, Web of Science, Cochrane Library, SciELO and LILACS databases, were searched for articles published from inception until February 2016. Two independent reviewers performed the quality assessment of the included studies. We performed a meta-analysis with random effects and inverse variance, and subgroup analyses were performed.

**Results:**

Ten trials involving a total of 1543 patients met the inclusion criteria. Our study showed reductions in cholesterol levels according to the intensity of statin doses (high, intermediate and low): (-104.61 mg/dl, -67.60 mg/dl, -56.96 mg/dl) and in the low-density lipoprotein cholesterol level: [-105.03 mg/dl (95% CI -115.76, -94.30), I2 19.2%], [-67.85 mg/dl (95% CI -83.36, -52.35), I2 99.8%], [-58.97 mg/dl (95% CI -67.83, -50.11), I2 93.8%. The duration of statin therapy in the studies ranged from 8 to 104 weeks, precluding conclusions about long-term effects.

**Conclusion:**

Statin treatment is efficient in lowering lipids in children with FH. There is need of large, long-term and randomized controlled trials to establish the long-term safety of statins.

## Introduction

Familial hypercholesterolemia (FH) is a dominant autosomal genetic disease. The worldwide prevalence is of 1 in 250 people affected with the heterozygous form (HeFH) of HF.^1^ FH is characterized by high levels of low-density lipoprotein (LDL) cholesterol due the reduced hepatic capacity to remove LDL-cholesterol from blood circulation,^2^ which can result in early atherosclerosis development.^3^ Further, children with FH have damage in the endothelial function and increased intima-media thickness (IMT)^4^ indicating early atherogenesis.

The hydroxy-methyl-glutaryl-CoA (HMG-CoA) reductase inhibitors or statins decrease the coronary morbidity and mortality in high-risk adults. They have proven to be effective in decreasing LDL-cholesterol levels and cardiovascular events in adults.^5^ Statins are one of the most prescribed drugs in the world^6^ for adults and, in usual doses, are notably safe.

The expert consensus recommends drug treatment for children older than 10 years old with LDL-cholesterol level ≥ 5 mmol/L (190 mg/dl), whose cholesterol levels remain elevated despite diet measures during the period from 8 weeks to 2 years for children ages 8-18 years. It is also considered the treatment for those with LDL-cholesterol ≥ 4 mmol/L (160 mg/dl) with the presence of two or more cardiovascular risk factors or family history of CVD.^2,7^

The US Food and Drug Administration (FDA)^8^ has approved the use of some statins like simvastatin, atorvastatin, fluvastatin, pravastatin, rosuvastatin and lovastatin for pediatric and adolescent patients. Pravastatin is approved for use at 8 years of age, other statins are approved for use from 10 years on*.* FDA^8^ recommends statins for children with FH, primary or genetic dyslipidemia. The treatment to reduce cholesterol levels in pediatric patients is based on evidence involving only adults.^9^ The effect of statins in pediatric population has been limited to short-term randomized clinical trials (RCTs).^10,11^

Thus, the aim of this study was to systematically review the literature to evaluate the effects of different statins and the dosages in elevated plasma levels of total cholesterol (TC), LDL- cholesterol and apolipoprotein B (APOB) and in decreased high-density lipoprotein (HDL) cholesterol levels in children and adolescents with FH. We also aimed to evaluate statin safety in this group.

## Methods

A systematic review was conducted according to Cochrane Collaboration and Preferred Reporting Items for Systematic Review and Meta-analyses: the PRISMA Statement.^12^

### Eligibility criteria

Studies included RCTs performed in children and adolescents from 8 to 18 years old, submitted to statin therapy for treatment of familial hypercholesterolemia. The intervention was considered as the use of statins at any dose, for at least eight weeks. Our protocol has assessed increased plasma levels of TC, LDL-cholesterol and APOB, and decreased HDL-cholesterol, in addition to seeking evidence on the effectiveness, safety and effects of statins. The RCTs were included if fulfilled the inclusion criteria and had at least one primary or secondary outcome. Studies that did not provide information on the magnitude of the intervention's effect in the control or experimental groups were excluded. When a study had several publications (or sub-studies), only the most recent was included. The other publications were used to supplement information.

### Information sources

The review protocol was registered in the International Register of Prospective Systematic Reviews (PROSPERO), under registration number: CRD42015029350. The search comprised seven online databases - PubMed, EMBASE, Bireme, Web of Science, Cochrane Library, SciELO and LILACS. It lasted from the beginning to February 2016 and was composed by entries related to the following terms: *"child", "adolescents", "cholesterol", "hypercholesterolemia", "statins", "dyslipidemia", "inhibitor hidroximethylglutaril-CoA reductase"*. There was no language restriction and we adopted a high-sensitivity strategy for the search of randomized controlled trials.^13^ To identify other primary studies, the authors searched and checked for reference lists of previously published systematic reviews and meta-analyses. The detailed strategies for PubMed are in Appendix I. The strategies for other databases are available upon request.

### Study selection and data extraction

Two investigators (G.R. and G.S.), in duplicate and independently, evaluated the titles and abstracts of all articles identified by the search strategy. The abstracts that provide enough information regarding the inclusion and exclusion criteria were selected for full-text evaluation. In the second phase, the same reviewers independently evaluated the full text of these articles and made their selection in accordance with the eligibility criteria. Disagreements between reviewers were solved by consensus, and when disagreement persisted it was solved by a third reviewer (L.C.P.). These two reviewers (G.R. and G.S.) independently conducted data extraction regarding the methodological characteristics of the studies, interventions and outcomes using standardized forms. The CONSORT analysis instrument was used to evaluate methodological quality (internal and external validation) of the included clinical trials. The outcomes extracted in this meta-analysis were: TC (mg/dl), LDL-C (mg/dl), HDL-C (mg/dl), APOB (mg/dl).

### Assessment of risk of bias

Quality assessment of studies included adequate sequence generation, adequate allocation concealment, blinding of investigator, participants, and outcomes assessors, intention-to-treat analysis and description of losses and exclusions. Studies had to have a clear description of an adequate sequence generation to fulfill these criteria. The description of how the allocation list was concealed could include terms like "central", "web-base" or "telephone randomization" or computer-generation.

Intention-to-treat analysis was considered as confirmation on study assessment that the number of participants randomized and the number analyzed were identical, except for patient lost to follow-up or those who withdrew consent for study participation. Two reviewers independently performed quality assessment, and, for each criterion, studies were classified as adequate, not adequate or unclear/not reported.

### Data Synthesis and Statistical Analysis

All analyses were conducted using Software RStudio.^14^ For continuous outcomes, if the unit of measurement was consistent throughout trials, results were presented as weighted mean difference with 95% of confidence intervals (CIs). Calculations were performed using random effects method and the statistical method used was inverse variance. Statistical significance defined for the analyzes as p < 0.05. Statistical heterogeneity of the treatment effects among studies was assessed using Cochran's Q test and the inconsistency I^2^ test. In addition, sensitivity analysis of RCTs was performed to assess differences in the intervention approach (intervention group versus placebo). In studies where statins therapy compared three different arms of treatment (intervention group) versus placebo (control group), we will conduct weighted average and divide the total number of patients to the distribution of the control group.^15^

## Results

### Description of studies

**Figure 1:**
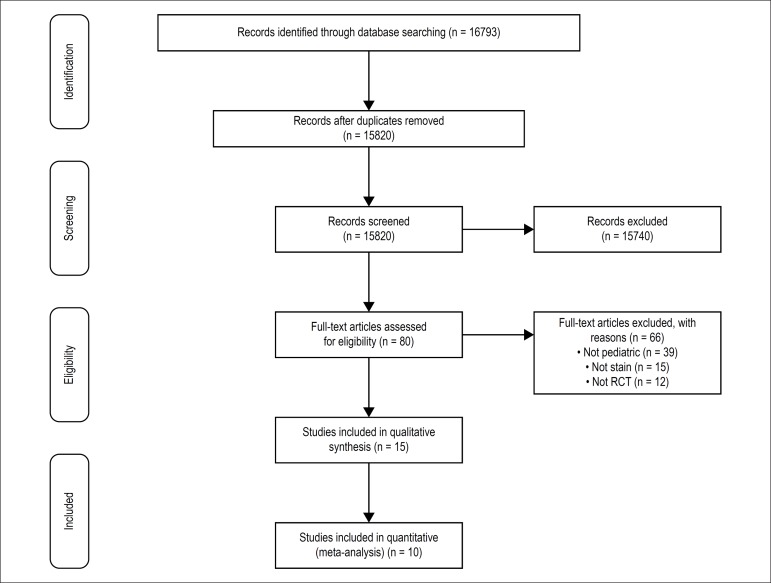
Summary of evidence search and study selection.

We initially identified 16793 potentially relevant citations from electronic databases. A total of 15 RCTs were included in the synthesis of qualitative studies and10 RCTs^10,11,16-23^ were selected to the quantitative analysis. Studies that were not eligible for the quantitative analysis did not provided data on cholesterol levels^24-27^ in a way that we could extract them from the article, and one study^28^ was not performed with a control group. Figure 1 shows the summary of evidence search and study selection in this review. The included studies comprised a total of 1543 subjects, and they were all full peer-reviewed publications.

### Participants

Table 1 summarizes the characteristics of participants and included studies. The number of participants in the studies ranged from 54 to 248. A total of 934 subjects received statin therapy and 609 received placebo. The age also varied from 8 to 18 years old. The studies have evaluated different types of statins for a period of 8 to 104 weeks.

**Table 1:** Characteristics of included studies

Study, year	Randomized patients (n) intervention/placebo	Participants Age range	Intervention group	Control group	Duration of intervention	Statistical significance	Evaluated outcomes
Knipscheer et al., 1996	54/18	8 to 16 years	Pravastatin: (1) 5 mg/day, (2) 10 mg/day, and (3) 20 mg/day	Placebo	12 weeks	p < 0.05	TC, LDL-C, TGs, HDL-C, apo A-I, apo B, Lp(a), VLDL-C, ALT, AST, hormones
Stein et al., 1999	67/65	10 to 17 years	Lovastatin 10 mg/day for 8 weeks; 20 mg/d for 8 weeks, 40 mg/day	Placebo	48 weeks	p < 0.05	LDL-C, TGs, TC, HDL-C, apo A-I, apo A-II, apo B, Lp(a), testicular volume, ALT, AST, hormones, growth and development
de Jongh et al., 2002	106/69	10 to 17 years	Sinvastatin 10 mg/day for 8 weeks; 20 mg/day for 8 weeks; 40 mg/day	Placebo	48 weeks	p < 0.05	LDL-C, CT, TGs, HDL-C, apo A-I, apo B, VLDL-C, hsCRP, ALT, AST, hormones
McCrindle et al., 2003	140/47	10 to 17 years	Atorvastatin 10 mg/day; 20 mg/day if LDL ≥ 3.4 at weeks 4	Placebo	26 weeks	p < 0.05	LDL-C, CT, TGs, HDL-C, apo A-I, apo B, ALT, AST, hormones
Wiegman et al., 2004	106/108	8 to 18 years	Pravastatin 20 mg/day if <14 years of age; 40 mg/day if ≥ 14 years of age	Placebo	104 weeks	p < 0.05	LDL-C, TGs, TC, HDL-C, Lp(a), carotid IMT, growth, maturation, hormone level, liver and muscle enzymes
Clauss et al., 2005	35/19	10 to 17 years	Lovastatin 20 mg/day for 4 weeks; 40 mg/day	Placebo	24 weeks	p ≤ 0.05	LDL-C, TGs, HDL-C, apo A-I, apo B, Lp(a), VLDL-C, ALT, AST, hormones
Rodenburg et al., 2006	90/88	8 to 8 years	Pravastatin 20 mg/day if <14 years of age; 40 mg/day if ≥ 14 years of age	Placebo	104 weeks	p < 0.05	LDL-C, TC, TGs, HDL-C, apo B, Lp(a), VLDL-C, carotid IMT, C-reactive protein, OxLDL markers, Immune complexes
	intervention/placebo	Age range			intervention		outcomes
Van der Graaf et al. 2008	126/122	10 to 17 years	Simvastatin: (1) 10 mg/day, 20 mg/day, or 40 mg/day plus ezetimibe 10 mg/day or placebo for 6 weeks; Sinvastatin: (2) 40 mg/day plus ezetimibe 10 mg/day or placebo for 27 weeks; All subjects received open-label: (3) simvastatin 10 mg/day or 20 mg/day plus ezetimibe 10 mg/day for 20 weeks;	Placebo	53 weeks	p < 0.05	LDL-C, TC, TGs, HDL-C, apo B
Avis et al., 2010	131/46	10 to 17 years	Rosuvastatin: 5 mg/day, 10 mg/day, 20mg/day	Placebo	12 weeks	p < 0.05	ALT, AST, CK, GFR, urine, TC, LDL-C, TGs, HDL-C, apo A-I, apoB
Braamskamp et al., 2015	79/27	6 to 17 years	Pitavastatin: 1 mg/day, 2 mg/day, 4 mg/day	Placebo	12 weeks	p < 0.05	TC, LDL-C, HDL-C, TGs, apo A-I, apoB

Abbreviations: hsCRP: high-sensitivity c-reactive protein, ALT: alanine aminotransferase, AST: aspartate aminotransferase, CK: creatine phosphokinase, apo B: apolipoprotein B, apo A-I: apolipoprotein A-I, apo A-II: apolipoprotein A-II, DHEAS: cortisol and dehydroepiandrosterone sulfate, FSH: follicle-stimulating hormone, LH: lutropin, IMT: carotid intima-media thickness, CK: creatine kinase, GFR: glomerular filtration rate; sPLA2: secretory phospholipase A2, TGs: triglyceride, VLDL-C: very low dfensity lipoprotein - cholesterol, LDL-C: low-density lipoprotein - cholesterol, TC: total cholesterol, HDL-C: high density lipoproteins - cholesterol, Lp(a): lipoprotein, Lp-PLA2: lipoprotein-associated phospholipase A2, OxLDL markers: oxidized low-density lipoprotein.

### Risk of bias in included studies

#### Allocation

##### Generation of sequence

The generation of the allocation sequence was adequate in two studies since the sequence was computer-generated.^10,18^ The remaining ten studies were described as randomized, but no further details of the process were given (Table 2).

**Table 2:** Risk of bias of included studies

Study, year	Adequate sequence generation	Allocation concealment^a^	Blinding of investigator	Blinding of participant	Blinding of outcome assessors	Intention - to-treat analysis^b^	Description of losses and exclusions
Knipscheer et al., 1996	Not reported	Unclear	Not reported	Not reported	Not reported	Yes	No
Stein et al., 1999	Not reported	Unclear	Not reported	Not reported	Not reported	Yes	Yes
de Jongh et al., 2002	Not reported	Unclear	Not reported	Not reported	Not reported	Yes	Yes
McCrindle et al., 2003	Not reported	Unclear	Not reported	Not reported	Not reported	Yes	Yes
Wiegman et al., 2004	Yes	Unclear	Not reported	Not reported	Not reported	No	Yes
Clauss et al., 2005	Yes	Adequate	Not reported	Not reported	Not reported	Yes	Yes
Rodenburg et al., 2006	Not reported	Unclear	Not reported	Not reported	Not reported	Yes	No
Van der Graaf et al. 2008	Not reported	Unclear	Not reported	Not reported	Not reported	Yes	Yes
Avis et al., 2010	Not reported	Unclear	Not reported	Not reported	Not reported	Yes	Yes
Braamskamp et al., 2015	Not reported	Unclear	Not reported	Not reported	Not reported	Yes	Yes

a Allocation concealment: Adequate (randomization method described that prevents caregivers or investigators from interfering or identifying before randomization; Unclear (randomization stated but no further information provided).

b Intention-to-treat analysis: Intention-to-treat and completeness of follow-up are assessed by results available at the end of trial. Yes (specified by authors and confirmed by our analysis), No (specified or not specified by authors but no evidence of intention-to-treat confirmed by our analysis).

##### Concealment of allocation

None of the included studies described how the allocation sequence was concealed from the investigators, the outcome assessors or the participants in the study (Table 2).

##### Blinding

All studies were described as double blind, indicating that participants and those participating in treatment procedures were blinded to treatment (Table 2).

##### Incomplete outcome data

From the studies included, 90% reported intention-to-treat analyses and 80% described losses due to follow-up and exclusions.

#### Effects of interventions

##### Statins versus placebo

All included studies describe the use of therapy with statins: atorvastatin,^16^ lovastatin,^10,21^ pravastatin,^17,18,19^ rosuvastatin,^20^ simvastatin^11,22^ and pitavastatin.^23^ The dosage and duration of treatment with statins varied between them (Table 1). The detailed analyzes are in Appendix II, III, IV, and V.

##### Change in Total cholesterol

**Figure 2:**
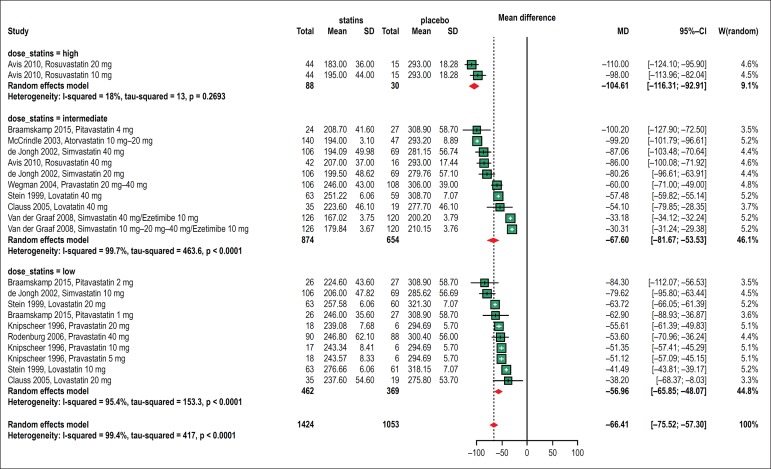
Forest plots showing the effect of statin therapy (high, intermediate and low dose) on total cholesterol (TC) levels.

Ten of the included studies evaluated the effect of statin therapy on the TC level.^10,11,16-23^ A subgroup analysis was performed in line with the intensity of statin doses, classified according to expected LDL-cholesterol reduction effect^29^: ≤ 30% as low; 30-40%, intermediate, and ≥ 40%, high. In this analysis, all subgroups maintained significant reductions in cholesterol levels (-104.61 mg/dl, -67.60 mg/dl, -56.96 mg/dl), and intragroup heterogeneity was lower (18%, 99.7%, 95.4%). This analysis explained 99.4% of the original heterogeneity found in the main analysis (Figure 2).

##### Change in LDL-cholesterol level

**Figure 3:**
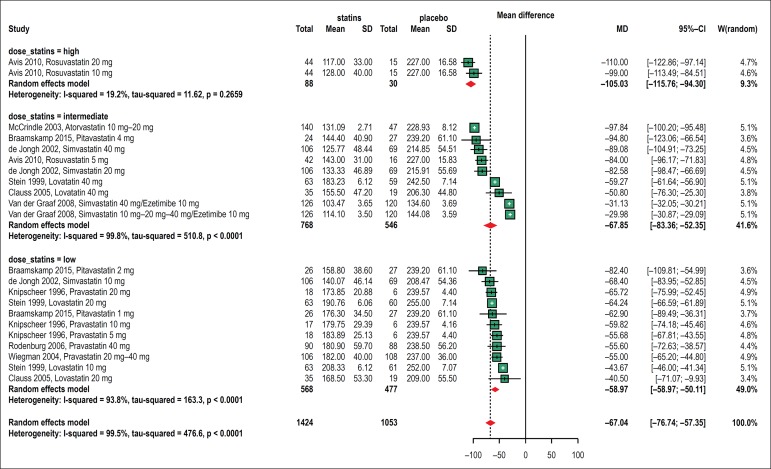
Forest plots showing the effect of statin therapy (high, intermediate and low dose) on low-density lipoprotein (LDL) cholesterol levels.

Ten included studies evaluated the effect of statin therapy on the LDL-cholesterol level.^10,11,16-23^ All subgroup analysis demonstrated significant reduction in this level: [-105.03 mg/dl (95% CI -115.76, -94.30), I^2^ 19.2%], [-67.85 mg/dl (95% CI -83.36, -52.35), I^2^ 99.8%], [-58.97 mg/dl (95% CI -67.83, -50.11), I^2^ 93.8%], (Figure 3). The detailed analyzes are in Appendices II, III, IV, and V.

## Discussion

We quantitatively analyzed ten randomized placebo-controlled trials in children with FH. Studies showed a clinically significant reduction in LDL-cholesterol levels in children treated with statin, compared to those treated with placebo. In addition, therapy with statins slightly increased HDL-cholesterol. The reduction in LDL-cholesterol levels varied between studies, probably due to different statins and dosages, and, possibly due to different settings of HeFH.

In our meta-analysis, the results of all studies using statins were combined. All statins included present a common mechanism of action, i.e., inhibition of hydroxy-methyl-glutary-Coa. All statins have shown beneficial effects in lowering lipid levels and have been approved for use in adult patients with dyslipidemia.

When comparing some results: the study using lovastatin to evaluate efficacy and safety in children, focusing on female population, concluded that the lovastatin group showed a reduction in LDL-cholesterol levels of 23% to 27% against an increase of 5% in the placebo group (p < 0.001), TC of 17% to 22%, and APOB of 20% to 23%.^10^ Whereas another study with young male patients,^21^ lasting 24 weeks, lovastatin significantly reduced LDL-cholesterol levels at all dosages compared with placebo (17%, 24%, 27% with dosage of 10, 20, and 40 mg/day, respectively; p < 0.001). Further treatment with the dose of lovastatin at 40 mg/day (from 24 to 48 weeks) reduced LDL-cholesterol by 25% compared to placebo (p < 0.001).

In a study with pravastatin, the assessed primary efficacy outcome was the IMT, showing a significant difference between pravastatin versus placebo (p = 0.02).^18^ Also, pravastatin reduced LDL-cholesterol levels (-24.1%) versus placebo (+0.3%) and p < 0.001. The authors suggest that IMT findings and efficacy of treatment with pravastatin in this study should be limited to children with FH.

The efficacy results of this study were similar to others. At the end of 48 weeks, patients treated with simvastatin showed statistically significant reductions in LDL- cholesterol levels (-41%), TC (31%), APOB (-34%), very low-density lipoprotein (VLDL) cholesterol (-21%) and triglycerides (TG) (-9%).^11^ In the study of atorvastatin versus placebo, there was an average reduction in LDL-cholesterol (40%), TC (32%), TG (12%) and APOB (34%) in the atorvastatin group compared to the placebo group (p <0.001). The increase in HDL-cholesterol levels (2.8%) was also statistically significant.^16^ In the study comparing rosuvastatin versus placebo, changes in LDL-cholesterol, TC, and APOB levels were statistically significant compared to placebo for all three doses (5 mg, 10 mg, 20 mg) (p < 0.001).^19^

Most of the studies included in this meta-analysis focused on the effect of statins on LDL. As seen in these results in children with FH, statins are effective in lowering LDL-cholesterol and TC levels. The effectiveness of reducing the LDL-cholesterol and TC levels with statin treatment is consistent in all RCTs analyzed. The effects of statins on other levels of lipids, such as HDL-cholesterol and TG are not so consistent; that is why the results are not extrapolated to the entire pediatric population. Patients without FH must focus on changes in lifestyle first, before relying on a drug to improve their cholesterol levels.

The included studies had essential elements that determine the quality of studies, which are important for the generation of evidence. Conducting a randomized controlled trial in the pediatric population is not as common as in adults. However, there is a lack of a recognized methodology to assess the quality of pediatric studies. That is the reason why we used the clinical testing format, as used in the adult population.

The adverse event profile of a pharmacological agent is a particular concern in pediatric population. Thus, in general, data suggest that the risk of adverse events in children treated with statins are similar to those observed in adults treated with statin, at least in the short term. Studies evaluated the effect of statin therapy on clinical outcomes, hormonal status, biochemical measures of growth, nutrition and liver or kidney toxicity. For most of these parameters, there was no statistically significant difference between treatment and placebo groups. There were no reports of serious adverse events. Hepatic transaminase elevation and Creatine-phosphokinase, which are of particular concern in adults, did not differ in the studied groups.

Current guidelines for FH indicate pharmacological treatment in affected subjects between 8 to 10 years and in younger children only with extreme elevation of LDL-cholesterol and associated risk factors, having risk for premature CAD.^30-33^ Statins can be considered as first line treatment in children with HeFH and having an increase of LDL, after changes in diet and lifestyle. Response to treatment with statins should be assessed in 1 to 3 months after the start of therapy and periodically thereafter according to guidelines.^34^ Children treated with statins should also be frequently monitored for adverse events (for example, hepatic transaminases, creatine kinase, liver enzymes) and statins are contraindicated during pregnancy.^34^ There is also a need for further studies to evaluate the safety of these pediatric patients throughout their lives. The results for the growth and sexual development should be considered in children under 10 years of age. Future studies should seek to include pediatric patients with secondary forms of dyslipidemia and start examining the combination of therapy in children.

However, we found some limitations in these studies. One of them is the duration of statin therapy in the included studies, which ranged from 8 to 104 weeks, whereas in the clinical practice, patients with FH are subjected to continue with statin treatment for the rest of their lives, once the therapy was initiated.^35^ Another limitation of these studies is the conduction only in children with FH and children with secondary dyslipidemia were not included.^35^ They also do not include information on the use of high doses of statins, such as those used in adults. Besides, the long-term efficacy data also are not available and remain unknown.

Braamskamp et al.^36^ published the first study evaluating hormonal concentrations of FH subjects before and 10 years after the start of treatment with statins, compared with their unaffected siblings, which minimizes genetic and environmental variation between groups. Their results demonstrated that the hormone concentrations in patients with FH are among the reference range compared to their unaffected siblings.

## Conclusion

Based on the evidence available in this meta-analysis, statins significantly reduced LDL-cholesterol in children with HeFH. However, there is no data regarding long-term outcomes of both effectiveness and safety.

## Figures and Tables

**Appendix 2:**
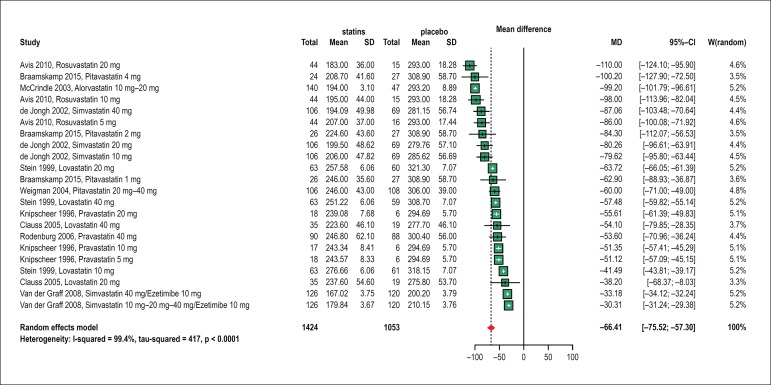
Forest plots showing the effect of statin therapy on total cholesterol (TC) levels.

**Appendix 3:**
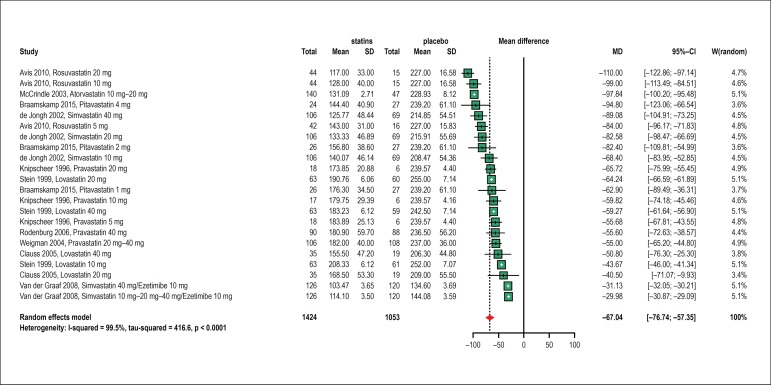
Forest plots showing the effect of statin therapy on low-density lipoprotein (LDL) cholesterol levels

**Appendix 4:**
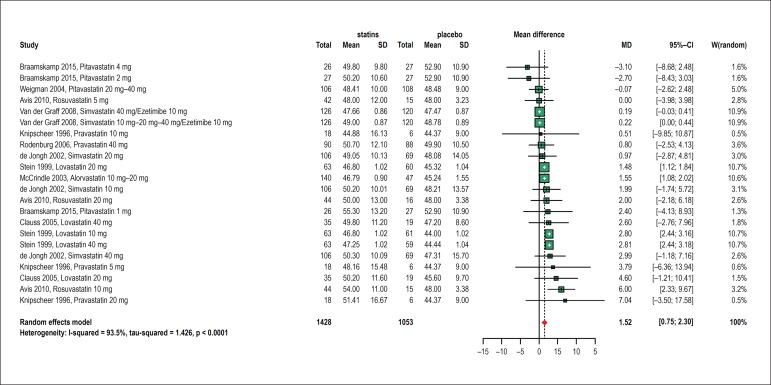
Forest plots showing the effect of statin therapy on high-density lipoprotein (HDL) cholesterol levels.

**Appendix 5:**
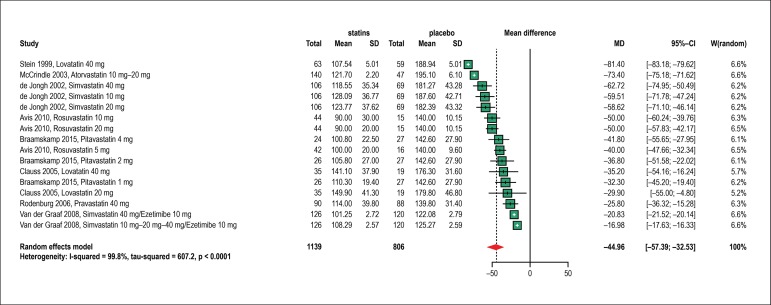
Forest plots showing the effect of statin therapy on apolipoprotein B levels.

## Appendix I

### PuBMed Search Strategy

#1. Search (Child OR Adolescent)

#2. Search (Hypercholesterolemia OR Statin OR Dyslipidemias OR Cholesterol OR Hydroxymethylglutaryl-CoA Reductase inhibitors)

#3. Search (randomized controlled trial[pt] OR controlled clinical trial[pt] OR randomized controlled trials[mh] OR random allocation [mh] OR double-blind method[mh] OR single-blind method[mh] OR clinical trial[pt] OR clinical trials[mh] OR ("clinical trial"[tw]) OR ((singl*[tw] OR doubl*[tw] OR trebl*[tw] OR tripl*[tw]) AND (mask*[tw] OR blind*[tw])) OR ("latin quare"[tw]) OR placebos[mh] OR placebo*[tw] OR random*[tw] OR research design[mh:noexp] OR comparative studies[mh] OR evaluation studies[mh] OR follow-up studies[mh] OR prospective studies[mh] OR cross-over studies[mh] OR control* [tw] OR prospectiv*[tw] OR olunteer*[tw]) NOT (animal[mh] NOT human[mh])

#4. Search (#1 AND #2 AND #3)
